# Dental Pathology and Surgery

**Published:** 1875-03

**Authors:** 


### BIBLIOGRAPHICAL.

*Dental Pathology and Surgery.*—By S. James A. Salter, M. B., F. R. S. Etc. Publishers: William Wood & Co., 27 Great Jones St., N. Y.

This is another very valuable contribution to dental literature, being a digested collection of the author's previous essays and papers arranged in the form of chapters, to which several not previously published have been added. The work is devoted more especially to Dental Surgery than to Dentistry proper, and the author claims his desire to be, as far as possible the exponent of authorized views and observations, or at least as the result of independent thought and investigation. The subjects treated are: General Anatomy of the teeth, Structure of the hard and soft tissues, Functions of the Teeth, Abnormal Teeth, Secondary Dentine, Congential Defects, Caries, and other Diseases of teeth, Odontomes, Diseases of Pulp, Tumors and Hypertrophy of the Gums, Dentigerous Cysts, Painful and difficult Eruption of Wisdom Teeth, Alveolar Abscess, Abscess of Antrum, Affections of Nervous System dependent on diseases of the teeth, Facial and General Neuralgia, Diseases of Alveolar Processes, Chemical character and composition of the Saliva, Extraction of Teeth, and Accidents resulting therefrom, and Defects of the Palate. The chapter on "Impaction of Permanent Teeth" is a very important and interesting essay, and the only omission in the work is that of the Development of the Teeth, which in our judgment, should have engaged the attention of the author. The work is embellished with one hundred and thirty three illustrations, and is gotten up in a style which reflects credit upon the publishers.

